# Exploring Practices During Nursing Handovers in Critical Care: An Anthropological Study

**DOI:** 10.1111/nicc.70389

**Published:** 2026-02-19

**Authors:** Stelios Parissopoulos, Fiona Timmins, Marianna Mantzorou, Meropi Mpouzika, Theodoula Adamakidou, Eleni Papagaroufali

**Affiliations:** ^1^ Department of Nursing University of West Attica Athens Greece; ^2^ Department of Social Anthropology Panteion University of Social and Political Sciences Athens Greece; ^3^ School of Nursing & Midwifery University College Dublin Dublin Republic of Ireland; ^4^ Department of Nursing Cyprus University of Technology Lemesos Cyprus

**Keywords:** clinical decision‐making, critical care nursing, ethnography, expertise, heterotopia, intensive care, nursing handover

## Abstract

**Background:**

Nursing handovers in intensive care units (ICUs) are critical for ensuring continuity of care, patient safety and clinical decision‐making. Beyond information transfer, handovers are key moments in which nursing expertise can influence treatment trajectories. However, power relations, communication practices and the visibility of nursing knowledge during handovers remain underexplored, particularly through the lens of critical medical anthropology and the concept of *heterotopia*.

**Aim:**

To explore how experienced ICU nurses participate in and influence clinical decision‐making through handover practices.

**Study Design:**

This ethnographic study, grounded in a critical medical anthropology approach, was conducted in a general ICU in Greece. Data collection involved overt participant observation and ad hoc ethnographic interviews with ICU nurses and physicians, as part of a larger PhD study. Nurses who participated in interviews subsequently consented to observation while working. Fieldwork spanned 2 years (2012–2014) and concluded in 2020. Multiple nursing handovers were observed, and thematic analysis was applied to fieldnotes using Atlas.ti.

**Findings:**

Nursing handovers functioned as *heterotopias of nursing expertise*, offering moments of access to clinical decision‐making. Four sub‐themes emerged: (a) narrating the patient's illness trajectory; (b) showcasing expertise and mentoring less experienced colleagues; (c) verbalising and consolidating clinical decisions; and (d) temporarily reversing hierarchical power dynamics. Through these practices, experienced nurses rendered their clinical reasoning visible and exerted influence over patient management.

**Conclusions:**

Nursing handovers represent strategic opportunities for nurses to navigate and renegotiate professional boundaries within the ICU. The findings highlight the potential of handovers to strengthen team collaboration, support clinical decision‐making and enhance patient care. Recognising handovers as protected spaces for nursing expertise is essential for promoting professional autonomy within critical care settings.

**Relevance to Clinical Practice:**

Protecting nursing handovers as spaces of professional dialogue can enhance communication, clinical reasoning and patient safety. Combining structured and narrative‐based handover training may empower nurses, preserve mentoring and role‐modelling functions and sustain professional visibility—particularly relevant in the post‐COVID‐19 era, where digital and hybrid communication models increasingly shape critical care practice.

## Impact Statements

- What is known about the topic?
  ○ Nursing handovers in intensive care units (ICUs) are critical for ensuring continuity of care, patient safety and clinical decision‐making.
  ○ Effective handovers contribute to better communication, reduce the risk of medical errors and support team cohesion.
  ○ However, power relations, communication patterns and the visibility of nursing expertise during handovers remain underexplored—particularly from an ethnographic and anthropological perspective that examines how culture and hierarchy shape these practices.
- What this paper adds?
  ○ This study conceptualises nursing handovers as *heterotopias*—protected spaces where nurses can assert their expertise and influence clinical decision‐making.
  ○ The ethnographic approach reveals how handovers provide moments of access to clinical decision‐making, enabling nurses to challenge traditional hierarchies and make their voices heard.
  ○ Findings highlight the enduring value of *face‐to‐face* handovers as spaces for mentorship, role modelling and informal learning—dimensions that risk being lost amid increasing digitalisation and ‘silent’ reporting.
  ○ The study underscores the potential of structured yet narrative‐based handover training to enhance team collaboration, strengthen clinical reasoning and improve patient outcomes.

## 1 Introduction

Nursing handovers are embedded in hospital culture and are essential for ensuring patient safety and continuity of care through the transfer of critical clinical information [1, 2, 3]. Practices vary across settings, underscoring the need for context‐sensitive approaches [4]. In intensive care units (ICUs), handovers play a particularly central role due to patient complexity, high acuity and the need for interdisciplinary coordination [5, 6]. They are typically detailed, systematic, and frequently conducted at the bedside, supported by monitoring technologies, electronic records and structured communication tools designed to enhance accuracy and safety [7, 8, 9, 10, 11].

Despite these advances, challenges persist in adapting handover practices to the dynamic and unpredictable ICU environment. Rapid changes in patients' conditions demand communication that is both structured and contextually sensitive. However, formal training in handover communication remains inconsistent, with many nurses relying on informal learning, resulting in variation in practice quality [12, 13]. Ineffective handovers have been linked to medication errors, treatment delays and preventable patient harm [14, 15, 16, 17], contributing to poorer outcomes and increased mortality risk [18].

Handover quality is also shaped by workplace culture. Negative organisational climates and low job satisfaction undermine communication, increasing interruptions, missed care and anxiety—particularly among less experienced nurses [2, 17, 19]. Beyond information exchange, handovers function as culturally embedded practices that influence team cohesion, supervision and professional learning [20, 21].

Building on debates about nurses' involvement in clinical decision‐making [22, 23, 24], this anthropological study examines handovers as moments where nursing expertise is asserted and contested. Employing ethnography to capture unwritten norms, sequential interactions and embedded power relations [25, 26, 27], the study explores how ICU hierarchies shape nurses' participation in decision‐making. While handovers are widely studied, few investigations address their cultural and political dimensions, which this paper seeks to illuminate.

## 2 Background / Justification for Study

Nursing handover is a collective clinical practice that supports continuity of care across hospital wards and intensive care units (ICUs) [28]. Handover practices include global, bedside, verbal and electronic reporting (WHO, [29]) and aim to convey clinically relevant information to ensure safe patient care [30]. In ICUs, handovers are typically detailed and systematic due to patient complexity, involving review of physiological data and ongoing treatments. While this supports continuity and clinical decision‐making, it can increase stress for less experienced nurses [2, 31]. Persistent challenges—such as time constraints, interruptions and missing information—highlight the need to understand how handovers balance structured communication with contextual flexibility in high‐acuity environments [32, 33, 34, 84].

Structured communication frameworks (Box 1) are widely implemented to standardise information exchange, reduce errors, and support shared mental models in healthcare teams [2, 7, 8, 9, 10, 11, 16, 35, 36]. Nevertheless, interruptions and environmental distractions remain significant barriers, with alarms, competing priorities and time pressure compromising communication quality [19, 37]. Organisational culture and hierarchical structures further shape nurses' willingness to speak up, influencing handover effectiveness and patient safety [37]. Although electronic records facilitate information sharing, reliance on digital systems alone may reduce the visibility of nursing knowledge, reinforcing the importance of verbal communication [38].

BOX 1 **Examples of structured mnemonics and formats for nursing handover.**

- **I PASS THE BATON**: Introduction, Patient, Assessment, Situation, Safety, THE Background, Action, Timing, Ownership, Next
- **SHARQ**: Situation, History, Assessment, Recommendations, Questions
- **Five P's**: Patients, Precaution, Plan, Problems, Purpose, and ‘ISBAR’ Introduction, Situation, Background, Assessment, Recommendation
- **SBAR**: Situation, Background, Assessment, Recommendation

The COVID‐19 pandemic intensified global attention on communication, teamwork and patient safety in critical care [37]. Under unprecedented pressures, effective handovers became essential to prevent errors and maintain continuity of care [39, 40]. Pandemic‐related constraints—such as isolation protocols and rapidly evolving guidelines—required ICUs to adapt communication practices, emphasising clarity, empathy and multidisciplinary coordination [41, 42, 43]. Infection control measures also renewed interest in structured and remote handover processes [44].

The ICU provides a distinctive setting to explore how nurses navigate trajectories of critical illness [45, 46, 47], assess patients through the medical gaze [48, 49, 50, 51] and participate in decision‐making during moments of access and exclusion (Schluter et al., 2011) [24, 85, 86]. While nursing handovers are widely studied, few have examined their cultural and social dimensions using an anthropological lens. As digital documentation and ‘silent’ handovers increase, concerns persist regarding the loss of mentorship, tacit knowledge and embodied communication [17, 19]. Understanding how expertise, visibility and power are enacted during face‐to‐face handovers remains critical as technology reshapes ICU communication practices [3, 38]. Building on previous work [52, 53], this study examines ICU handovers as heterotopic spaces where nursing expertise becomes visible and professionally consequential.

## 3 Aims

To explore if and how experienced ICU nurses participate and influence clinical decision‐making through handover practices in Greece.

## 4 Design and Methods

### 4.1 Design

This ethnographic study drew on critical medical anthropology [48, 54] and phenomenology [55] to examine ICU nursing practices, with particular attention to handovers as dynamic, time‐bound interactions. A critical ethnographic orientation was used to question power relations and professional hierarchies in clinical decision‐making [56, 57]. Handovers were analysed as moments of access to decision‐making [24] and, drawing on Foucault's concept of heterotopia (1984/1967), also as protected spaces of professional visibility (Box 2).

BOX 2 **Preparing to enter the field.**

**Fieldnote entry—reflective comment**:

The decision to conduct my ethnographic study in this ICU was shaped by my professional background and my established rapport with the unit. As a nursing lecturer supervising student placements, I had already built trust with the nursing staff, which facilitated my immersion in the field. My experience as an ICU nurse allowed me to recognise subtle aspects of nursing practice while maintaining an ethnographic perspective.

Prior to entering the field, I engaged in reflective writing and theoretical reading to identify potential biases and to interpret everyday practices through a critical lens. Once in the field, ICU nurses shared their clinical routines, experiences and emotions, revealing a broader cultural ethos that shaped what it meant to be an ‘ICU nurse’. However, professional identities are not confined to a single space but extend across multiple contexts [58]. While the ICU remained my primary site, I also engaged with nurses involved in research and teaching, ensuring that my interpretations situated the Greek ICU context within wider social and cultural frameworks.

### 4.2 Setting and Sample

This study was conducted in a general ICU of a public hospital in Athens, Greece. The unit operated 15–18 adult beds, with a 1:3 nurse‐to‐patient ratio. The patient population comprised individuals requiring intensive care for medical, surgical or trauma‐related conditions. Staffing included physicians, intensive care specialists, permanent and temporary nurses and nursing and ward assistants. During the national financial crisis, staffing shortages led to fluctuating bed capacity, increased workloads [59, 60, 61] and missed nursing care [62, 63, 64], shaping daily routines and handover practices examined in this study.

### 4.3 Data Collection Tools and Methods

Data were collected over 2 years (2012–2014) as part of a doctoral project completed in 2021. The study began with semi‐structured interviews with experienced ICU nurses focusing on professional roles, clinical decision‐making and lived experience. All participants provided written informed consent for interviews and subsequent observation during clinical shifts; only those consenting to both phases were included in the observational component. Overt participant observation, a core ethnographic method [65], enabled the examination of ICU cultural practices from within. This approach captured both formal procedures and informal, often invisible, dimensions of work, including unwritten norms, power relations and professional hierarchies. Sustained immersion allowed observation of handovers as they unfolded in real time, shaped by clinical urgency, spatial constraints and interpersonal dynamics. Reflexivity was integral to the study; the principal investigator maintained a reflective journal and engaged in regular supervision to balance insider knowledge with analytic distance. For this paper, analysis focuses on observed nurse‐to‐nurse handovers as ethnographic episodes, illuminating how communication, clinical knowledge and professional identity are enacted and negotiated within the ICU.

### 4.4 Timeline of the Study and Relevance

Fieldwork was conducted between 2012 and 2014, with analysis completed in 2020, reflecting the iterative and interpretive nature of ethnographic research in complex institutional settings such as ICUs [65]. Although formal fieldwork ended in 2014, the researcher remained professionally connected to the unit, confirming that handover routines and professional dynamics have remained stable through 2019 and continue today (2025). Recent global attention to communication and teamwork in critical care following COVID‐19, alongside growing concerns about digital and ‘silent’ handovers, underscores the ongoing relevance of examining face‐to‐face handovers as sites of expertise sharing and mentorship [17, 19, 37].

### 4.5 Data Analysis

Data were analysed inductively and organised into patterns and themes using Atlas.ti v8. Braun and Clarke's [66] six‐phase thematic analysis guided the process: familiarisation, coding, theme development, review, naming and reporting. This flexible approach, well aligned with phenomenological inquiry, supported systematic interpretation of qualitative data [67, 68, 69]. Thematic analysis enabled the grouping and theorising of data—sometimes described as ‘grounded theorising’—to facilitate meaningful interpretation and analytic sense‐making. Ethnographic observations enriched the analysis by contextualising interview material and illuminating situated practices. For the purposes of this paper, analysis draws primarily on observational data. Fieldnotes were expanded into ethnographic scenes and systematically organised and coded within Atlas.ti v8, supporting an integrated and rigorous analytic process.

### 4.6 Rigour—Trustworthiness

Trustworthiness was established through credibility, confirmability, dependability and transferability [68, 70, 71]. Credibility was enhanced through 2 years of prolonged field engagement and data triangulation, including interviews, participant observation and consultation with experienced ICU nurses, situating findings within the wider healthcare context of Greece. Transferability was supported through purposive sampling, while confirmability and dependability were ensured via reflexive journaling, detailed documentation of analytic decisions and member checks. Ethnographic research is inherently time‐intensive due to the volume and complexity of data produced. Its iterative analytic process—moving between data and theory—allows for the identification of tacit knowledge and the generation of nuanced theoretical insights that are often inaccessible through other methodological approaches [65].

### 4.7 Ethical and Institutional Approvals

Although the study did not involve patients or vulnerable populations, it adhered to the ethical principles of the Declaration of Helsinki, including voluntary participation, informed consent and confidentiality. Ethical approval was obtained from the doctoral university programme and the Institutional Research and Ethics Committee of a public general hospital in Athens (protocol no 7/11‐7‐2012). ICU staff were informed of the study's purpose and the voluntary, overt nature of participation. Nurses who participated in interviews provided written informed consent for both interviewing and subsequent observation during clinical shifts; only those consenting to both phases were included. Participants were informed of the researcher's role, reminded of their right to withdraw and were observed with minimal disruption. Pseudonyms were used, and no patient data were collected. All personal names used in quotations and in the findings section are pseudonyms.

## 5 Findings

Nursing handovers in this ICU emerged as pivotal moments in which nurses actively participated in clinical decision‐making. The overarching theme identified was that handovers functioned as heterotopias of nursing expertise—temporary moments of access that enabled experienced nurses to articulate clinical knowledge and influence patient care (Table 1). Four interrelated sub‐themes were identified: (A) narrating the patient's illness trajectory; (B) showcasing expertise and mentoring less experienced colleagues; (C) verbalising and consolidating clinical decisions; and (D) reversing power dynamics within the unit. Drawing on Foucauldian notions of access and exclusion, handovers contrasted with the highly regulated routines of ICU life by offering nurses a protected space to assert expertise and shape clinical priorities.

**TABLE 1:** Heterotopia of nursing expertise: Overarching theme, sub‐themes and code categories.

Overarching theme	Sub‐themes	Code categories
Heterotopia of nursing expertise*—a moment of access enabling experienced nurses to contribute to patient care decisions*	(A) Narrating the patient's illness trajectory	Experienced nurses: Producing systematic descriptions of patient clinical condition. Novice nurses: missing information and being less systematic in their narrations. Prioritising the biological profile of the patient. Passing on key information and tasks to the next shift. Using and consulting effectively the patient observation chart. Panoptism of the body. ICU and medical terminology/jargon. Lack of global report in this icu.
	(B) Showcasing expertise and mentoring less experienced colleagues	Checking on the less experienced nurses. Offer advice and highlight issues of concern. Teaching the less experienced nurses. Identifying missed care.
	(C) Verbalising and consolidating clinical decisions	Justifying and consolidating clinical decisions that took place in the shift. Questioning clinical decisions. Initiating or considering new decisions.
	(D) Reversing power dynamics within the unit	Nursing report: ritual, specific format per unit. Spatial practices: taking up bedside space. Temporal dimension: protected time allocated for nursing reports: no visits, no phonecalls, no interruptions. Nurses being visible as a cohesive team. Controlled participation in the handover process.

In this unit, global handover reports were omitted in favour of in‐depth bedside handovers. Each nurse handed over responsibility for three patients to the incoming colleague. Verbal reports lasted approximately 15–20 min and concluded with the departure of the outgoing shift. These exchanges unfolded amid a dense sensory environment: the continuous alarms of monitoring equipment, ringing phones, background radio noise, administrative work by physicians at the nurses' station and moments of laughter and informal interaction among staff.

Handovers focused predominantly on anatomy and physiology, emphasising the maintenance of homeostasis and survival. Patients were often referred to through condensed clinical descriptors—such as ‘a cardiac arrest’, ‘a liver’ or ‘a head’—which signalled key priorities related to monitoring, interventions and equipment needs. Although used informally and sparingly, this shorthand reflected a practical orientation towards the critically ill body, foregrounding biological function over biography during this acute phase of care.

Typically, the outgoing nurse led the handover narrative, structuring the presentation of clinical information. However, receiving nurses frequently interrupted with clarifying questions, transforming the report into an interactive dialogue. This question‐and‐answer format facilitated the exchange of both explicit information and tacit knowledge, reinforcing handovers as collaborative encounters where nursing expertise became visible, negotiated and consolidated within the interdisciplinary context of the ICU.

### 5.1 Sub‐Theme A: Narrating the Patient's Illness Trajectory

This sub‐theme captures how nurses narrated patients' clinical conditions during handovers, revealing clear differences between experienced and less experienced nurses in structure, content and clinical reasoning. Experienced nurses delivered systematic, detailed accounts that prioritised each patient's biological profile and current physiological status. Their narratives were closely guided by the patient observation chart, which functioned as an extension of the clinical gaze, enabling them to select, organise and communicate key events relevant to continuity of care. Technology—monitors, infusion pumps, alarms, live physiological data and medication charts—played a central role in shaping these accounts, producing transparent, data‐rich handovers that facilitated safe transitions between shifts.

In contrast, novice nurses often struggled to maintain a coherent and structured narrative. Their reports were more fragmented, occasionally omitting information critical for understanding the patient's overall condition, such as the initial reason for ICU admission, significant clinical events or changes in trajectory. This disparity highlights handovers as both clinical and pedagogical encounters, where reporting practices simultaneously transmit patient information and reveal levels of professional competence.

Each bedside exchange served as the sole source of information for incoming nurses, as there was no unit‐wide overview provided by senior staff. As a result, the quality of individual narratives became particularly important. As one experienced nurse explained:

**Ethnographic interview—Nurse Anaxagoras (pseudonym)**: ‘I can tell from the way a person hands over the patient what kind of shift they've had—and what kind of nurse they are. I mean, whether they are organised and up to date with what needed to be done’.

Handovers reflected a form of panoptic practice, where the body was continuously surveilled and assessed through biomedical indicators (Box 3).

BOX 3 **Bedside handover to oncoming Nurse Litsa.**

**Fieldnote entry**:

During a bedside handover at the end of the morning shift, Artemis and Akrivi, both experienced ICU nurses, handed over their patients to Litsa, who was assuming responsibility for three patients for the afternoon shift (in line with the unit's 1:3 nurse‐to‐patient ratio). Artemis, the shift coordinator, spoke quickly but clearly, **with a** steady and confident voice and **a** relaxed body posture that reflected her clinical experience. Her familiarity with her patients was evident as she narrated their conditions, demonstrating her expertise and authority.

Drawing on Foucauldian notions of power circulating through knowledge, experienced nurses like Artemis asserted authority through clinical clarity and structured communication. In one observed handover, Artemis carefully guided her colleague Litsa through the patient's condition using the observation chart, emphasising potential challenges for the upcoming shift. Importantly, physicians present in the unit refrained from intervening, allowing the nursing handover to unfold uninterrupted (Box 4). Akrivi was equally capable of reporting her patient with clarity, but lacked the confidence of Artemis.

BOX 4 **Nurse Akrivi reports on her patients.**

**Fieldnote entry**:

Similarly, though less experienced, Akrivi conveyed the critical information Litsa needed, focusing on changes in each patient's clinical condition. However, she also highlighted a piece of information that she had forgotten to relay to the physicians during the morning round, reinforcing the importance of accurate and timely communication. Both nurses' narrations prioritised biological information essential for maintaining homeostasis, with minimal reference to the patients' personal identities or family contexts. For example, Akrivi stated, ‘patients at Four [referring to bed number four] has an IV, is unfed, his diuresis is good’—a phrasing that underscored the biomedical focus characteristic of ICU handovers.

Overall, this sub‐theme illustrates how experienced nurses used structured narration, technology and clinical language to shape understanding, reinforce expertise and maintain continuity of care at the bedside.

### 5.2 Sub‐Theme B: Showcasing Expertise and Teaching the Less Experienced

This sub‐theme highlights how senior nurses used handovers as pedagogical spaces to mentor less experienced colleagues through checking work, offering advice and identifying missed care. These interactions aimed to safeguard patient safety and promote learning, though they sometimes generated anxiety among junior nurses who feared criticism. An afternoon handover in which senior nurse Duchess took over patients from Elsa and Elpiniki illustrates this dynamic (Box 5).

BOX 5 **Senior ICU Nurse Duchess is taking over her patients at the start of the afternoon shift.**

**Nurses Elsa and Elpiniki handing over their patients:**

**1st patient**

– Elsa described diuresis levels, IV fluids, and blood sugar levels. Observing the monitor, Duchess questioned, ‘Only 92% saturation?’ to which Elsa replied, ‘Up to 95%. He has secretions’. Nurse Duchess acknowledged with a brief, ‘Yes’.

**2nd patient**

– Elsa reported hemodynamic instability, reduced urine output, and recent removal of an arterial line. Duchess interrupted, ‘Doesn't he have an [arterial] line?’ Elsa explained, ‘They took the line out. We're holding pressure at 120 mmHg, with diuretic half‐dose three times daily. If urine is up to 200ml, we hold Lasix; if not, we give it. Propofol is at 10 ml/hour. He had two IV bags stat, with slow pupil response to light’. Duchess questioned the target pressure, stating, ‘Isn't it the mean pressure that counts and not the systolic?’ Elsa responded, ‘That's what the physician told me—do you want me to ask her again?’ Duchess replied, ‘I'm not arguing with what you're saying’. The exchange became tense as Elsa, visibly frustrated, continued her report without further interruption.

**3rd patient**

– Elpiniki reported respiratory distress and recent extubation: ‘She's on a Venturi mask at 60%, saturation is stable, last sugar was 212’. Duchess asked, ‘Is this her normal? Shortness of breath like this?’ Elsa intervened, ‘She was more awake earlier, but she's a psychiatric case. She doesn't talk’. Duchess questioned, ‘Wasn't she on BiPAP?’ Elsa replied, ‘No, not today. She was intubated yesterday, but we don't know if the lithium affected her consciousness’. Elpiniki added, ‘Intubation prep is ready if needed’. Duchess observed, ‘She needs suction via her nose’. Elpiniki admitted, ‘I didn't suction her’. Duchess ended the handover with, ‘That's enough for me’. She then scanned the patients' charts, checked ventilators and IV pumps, and, dissatisfied with the patients' conditions, approached the physicians to request reassessments. The next day, Patient 3 was reintubated following respiratory failure.

During the handover, Duchess questioned clinical decisions, scrutinised physiological parameters, and highlighted areas where escalation may have been delayed. Her direct questioning revealed how authority and mentorship coexist uneasily in critical care. While the interaction was experienced as stressful, several junior nurses later reflected that such encounters—though uncomfortable—were pivotal for developing clinical judgement and professional accountability. Handovers thus functioned as informal training moments where expertise was both demonstrated and transmitted, reinforcing the role of experienced nurses as role models within the ICU team.

### 5.3 Sub‐Theme C: Verbalising and Consolidating Clinical Decisions

This sub‐theme focuses on how handovers enabled nurses to verbalise, justify and consolidate clinical decisions made during the shift. Nurses explained their reasoning, evaluated outcomes, and considered further actions, reinforcing their role as active decision‐makers. This process reinforced nursing expertise in decision‐making and highlighted nurses' roles as critical contributors to patient care. The handover by Nurse Voula, an ICU nurse with 2 years' experience, illustrates this process (Box 6).

BOX 6 **Observing Nurse Voula reporting on her patients at the end of her morning shift.**

**1st patient**

– **Voula**: ‘For Mr ___, I have nothing to report. He's stable, with good diuresis, on Lasix on a regular basis, his blood glucose is normal, and I managed to remove a lot of secretions with suctioning’.

**2nd patient**

– **Voula**: ‘Haemodynamically, he goes up and down—between 5 and 7 ml/hour [inotrope rate]. Right now, it's at 9 ml. At 12:45, he developed tachycardia, but he's back in sinus rhythm now. He had 55 pulses and a blood pressure of 95 mmHg. I was about to prepare Angoron, but he stabilised’.
– **Receiving nurse**: ‘He has a lot of secretions and fluctuating oxygen saturation, ranging from 93% to 96%’.
– **Voula**: ‘I informed the physician earlier. He had an X‐ray this morning, but I'm not sure whether they've reviewed it. After suctioning, his saturation was fine. His temperature reached 37.8°C. His urine output had dropped to 45 ml/hour, so we started diuretics on a regular basis. The right drain had only a few drops, while the left drained about 80 ml. Enteral feeding via the Levin tube is around 80 ml’.
– **Receiving nurse**: ‘What is he *drinking*?’ (i.e., what IV fluids is he on?)
– **Voula**: ‘One IV infusion of normal saline at 60 mL/h. I've added [name of drug] to the CVP line and left a free IV cannula available for medications’.

**3rd patient**

– **Voula**: ‘He's stable, but he wasn't passing urine, so I performed a bladder washout and administered 1,000 ml of IV fluids’.

Voula articulated the rationale behind interventions such as adjusting inotrope infusion rates and performing a bladder washout in response to reduced urine output. She also acknowledged unresolved concerns, including fluctuating oxygen saturation, enabling continuity of care. Importantly, handovers were often the only moments where nurses' clinical decisions became visible to colleagues. Unlike written records, which rarely captured nurses' suggestions or initiated changes, verbal handovers provided a platform to articulate judgement, expertise and responsibility. Through this process, nursing knowledge was legitimised, shared and integrated into ongoing patient management.

### 5.4 Sub‐Theme D: Reversing Power Dynamics in the ICU

One of the most striking findings of this study was how nursing handovers temporarily disrupted established hierarchical power relations within the ICU. During handovers, nurses symbolically and physically occupied bedside spaces, creating a protected temporal and spatial ritual that reinforced their presence as knowledgeable clinicians and decision‐makers. This dialogical interaction supported the transfer of clinical information and facilitated the negotiation of expertise within the nursing team. Across fieldnotes, the preservation of physiological homeostasis emerged as a unifying clinical priority, shaping both the content and delivery of the handover.

Handovers were safeguarded against interruptions, creating a protected time in which nurses could focus solely on clinical discussion. This temporal enclosure transformed reporting into a performative act through which nurses articulated clinical observations, justified decisions and advocated for patient needs, subtly influencing medical trajectories (Box 7). Rather than functioning as routine exchanges, handovers became moments of heightened professional visibility and authority.

BOX 7 **The use of abbreviations and medical terminology.**

**Field note entry**:

As I stood by as a silent witness to the verbal report, I noticed how nurses consistently used abbreviations and medical terminology when presenting their cases. They delivered their narratives casually yet confidently and, at times, dramatically, in order to emphasise critical pieces of information. The specialised language they employed constructed a narrative for each patient, transforming observation charts filled with measurements, numbers and clinical data into coherent clinical stories.

It became evident that in critical care, the physical body takes precedence—family and social history recede into the background. As I noted: ‘We don't see relatives a lot; they come later, just for half an hour, between 15:00 and 15:30 in the afternoon. This is when they claim their beloved one back from the ICU team’.

Spatial positioning further reinforced this shift. Standing together at the bedside enabled direct patient assessment while simultaneously marking nursing presence within an environment typically dominated by medical authority. The collective stance of nurses during handovers strengthened their identity as a cohesive team, contrasting with the more individualised positioning of physicians during ward rounds (Box 8).

BOX 8 **The shrinking *face* of the patient.**

**Field note entry:**

During nursing handovers, discussions focused predominantly on anatomy and physiology, prioritising the biological regulation of the body over the patient's broader personhood. I repeatedly observed how the patient's ‘face’—their identity as a person—seemed to recede during this critical phase of illness. In these moments, the patient was reconstituted through clinical narration: transformed into parameters, medications, drains and targets for intervention.

The human element did not disappear entirely but was temporarily overshadowed by the urgency of maintaining homeostasis and preserving life. This reduction was not careless; rather, it reflected the cultural logic of intensive care, where survival takes precedence over biography.

As an observer, I came to understand this narrowing of focus as both a professional necessity and a culturally sanctioned way of ‘seeing’ the critically ill body.

An example is illustrated in the following field note excerpt:

**Gregoris handing over a patient to Artemis:** Gregoris speaks quickly and succinctly as they move together along the foot of each bed.

– ‘Angoron running… fentanyl… dormicum—we need to change it so we don't keep replacing it… Bülau, Bülau—drain, drain, drain…’ *(He gestures towards the drains; Artemis counts them one by one.)*

The quality and structure of handover narratives varied according to the experience of the reporting nurse. Experienced nurses delivered more systematic and coherent reconstructions of patients' illness trajectories, effectively narrating the biomedical profile of each case. These reports not only prepared colleagues for the incoming shift but also produced clinically meaningful knowledge that could shape subsequent medical decisions. For the duration of the handover, informal nursing judgements became narratively visible, and expertise shifted from latent to enacted (Box 9).

BOX 9 **The sensory and organisational context of verbal handover in the ICU.**

**Fieldnote entry**:

The ritual and context of verbal handovers were surrounded by a symphony of activity. Equipment alarms sounded continuously, phones rang persistently, physicians sat at computer stations prescribing medications and the soft murmur of nurses' conversations formed the backdrop. There were no ward clerks employed in the unit.

Amidst this, nurse Voula handed over her patients with precision: ‘The gastrostomy is still leaking around… [name of patient] is stable, he communicates well, eats well…’ As Voula continued, the receiving nurse moved on to speak with physicians, drawing attention to a concerning drop in her patient's blood oxygen saturation and questioning whether a return to ventilator support was needed.

Responsibility for the patient was formally transferred upon completion of the handover. Its content, structure and duration reflected nurses' clinical experience and shaped priorities for the upcoming shift. These moments enabled clarification of earlier decisions and laid the foundation for new ones, reinforcing the handover as both a clinical and strategic site of practice.

These observations highlight the dual nature of nursing handovers: they ensured continuity of care while simultaneously functioning as performative spaces where nurses asserted professional judgement and negotiated power. In Foucauldian terms, the handover functioned as a heterotopia—a space that both mirrors and temporarily disrupts dominant norms [73]. Within this *heterotopia*, the temporal dimension seemed both magnified and slowed down, allowing nurses to become ‘lost’ yet fully present in the world of their patients. For the duration of the handover, this space could reverse, contest, suspend and neutralise the established power dynamics of the ICU. The *heterotopia* of the nursing handover activated a transition of nurses into a state of visibility and professional presence, effectively blunting the rigid power relations that typically governed the ICU environment.

## 6 Discussion

This study contributes to the literature on nursing handovers in critical care by demonstrating how handovers function not only as mechanisms for continuity of care, but also as pivotal moments of access and visibility for ICU nurses within hierarchical clinical environments. Our findings show that handovers create opportunities for nurses to participate meaningfully in clinical decision‐making, often through subtle yet influential practices. These results align with previous work highlighting the role of handovers in supporting decision‐making, professional communication and patient safety [4, 72].

One key contribution of this study is the conceptualisation of nursing handovers as a Foucauldian heterotopia. Drawing on Foucault's notion of heterotopia as a real yet distinct space that temporarily inverts institutional orders [73, 74], handovers in this study are understood as spaces and moments within the ICU where nurses gain visibility, assert expertise and influence clinical decision‐making. These ritualised spaces operate through controlled access and temporal isolation, enabling focused clinical discourse and the reconfiguration of professional power relations.

Ethnographic data showed that handovers were not merely informational exchanges but deeply performative practices. Experienced nurses used handovers to teach less experienced colleagues, validate or question clinical decisions and advocate for patient needs. These findings support previous research identifying handovers as sites for education, risk management and team cohesion [20], UK, and extend this literature by showing how the performance of expertise during handovers contributes to the construction of professional identity. Through verbal narration, spatial positioning and control of the interaction, nursing expertise shifted from being tacit to being publicly recognised, reinforcing group cohesion and professional confidence [17], China.

The concept of ‘moments of access’, as described by White et al. [24], provides a useful lens for interpreting these dynamics. Moments of access refer to temporary openings within institutional spaces where practitioners move between invisibility and visibility. In this study, handovers functioned as strategic moments of access, enabling experienced nurses to influence care trajectories through ordering, reframing or questioning clinical priorities. These moments allowed nurses to engage in clinical discourse that is often dominated by medical staff, consistent with findings by Manias and Street [75] and Coombs [76], who highlighted the negotiation of power and professional credibility in ICU practice.

Our findings further demonstrate that, despite the widespread use of structured handover tools such as ISBAR [77], the cultural and situational context of each ICU remains central to how handovers unfold. While standardised tools enhance consistency and safety, they may constrain narrative expression [37], Australia. In contrast, the self‐scripted narratives delivered by experienced nurses during handovers enabled nuanced transmission of clinical reasoning and supported continuity of care. This aligns with Fealy et al. [78] and Jeffs et al. [13], who emphasised the value of narrative practices in fostering critical thinking and shared understanding.

Handovers also emerged as moments where nurses navigated institutional constraints. Although ICU routines often functioned as mechanisms of control, handovers provided nurses with opportunities to temporarily occupy critical spaces and articulate their clinical judgement. This supports earlier work indicating that nursing practice frequently involves negotiating institutional structures to optimise patient care [78], Ireland; [23], Australia. In this sense, handovers operated as micro‐political arenas in which nurses asserted influence over clinical decisions traditionally governed by physicians [21], UK.

Seminal studies have long acknowledged the dual role of handovers in reinforcing both professional hierarchies and team cohesion [79], UK; [32], UK. This study builds on that foundation by demonstrating how the heterotopic quality of handovers enables a temporary inversion of power relations, allowing nursing voices to emerge more prominently within the ICU. The quality and structure of handover narratives are closely linked to clinical experience, with experienced nurses constructing more comprehensive accounts that could shape subsequent clinical decisions.

Evidence suggests that formal training in handover communication improves decision‐making and patient safety [12], UK; [28], Australia. Our findings support the importance of such training, particularly when combined with role modelling by experienced nurses. While structured tools benefit novice nurses, the performative and relational aspects of handovers remain critical for professional development and accountability.

Finally, the increasing shift towards electronic handover tools [80, 81] and remote handovers [82]—accelerated during the COVID‐19 pandemic—raises concerns about the erosion of pedagogical and role‐modelling functions embedded in face‐to‐face handovers. This study demonstrates that oral handovers are not merely transactional but function as embodied spaces of mentorship, judgement and collective reasoning. Emerging evidence suggests that digital or ‘silent’ handovers may reduce opportunities for informal knowledge sharing and diminish the visibility of nursing expertise [38]. While hybrid approaches may mitigate these effects, our findings underline the continued relevance of face‐to‐face handovers in ICUs today. By documenting these practices ethnographically, this study offers timely insights into what must be preserved as communication becomes increasingly technologised.

### 6.1 Limitations

A key strength of this study lies in its ethnographic design, which enabled a deep understanding of the cultural, social and clinical context of nursing handovers in a Southern European ICU, in Greece. Prolonged field engagement enhanced credibility, while the combination of participant observation and interviews enriched data robustness [68, 70]. Limitations include its single‐site focus and the data's pre‐pandemic timeframe, which may limit transferability [71]. Nonetheless, given that face‐to‐face handovers continue to characterise critical care globally, the findings remain relevant. Reflexive journaling and supervisory oversight helped mitigate potential bias linked to the researcher's dual role as insider and observer [68]. Integrating critical medical anthropology and phenomenological perspectives—particularly through Foucault's (1984/1967) and White et al.'s [24] concepts of heterotopia—offers an enduring interpretive framework for understanding how nursing practice evolves amid shifting institutional and technological landscapes.

## 7 Implications for Practice

This study highlights handovers as critical spaces where nursing expertise, judgement, and professional identity are enacted, underscoring the need to protect face‐to‐face handovers as integral to safe critical care practice [4]. While structured frameworks such as SBAR or ISBAR enhance consistency [77], they should complement rather than replace the narrative dimensions that support clinical reasoning and shared understanding [20]. As electronic, digital and hybrid handover systems expand, preserving the pedagogical and relational functions of oral handovers remains essential for knowledge transfer and mentorship [17, 38]. Targeted, simulation‐based handover training integrating structured and narrative communication may further strengthen continuity of care [83].

## 8 Conclusion

This study demonstrates that nursing handovers in critical care are more than procedural exchanges; they are performative practices through which nursing expertise becomes visible and influential within ICU hierarchies. Conceptualising handovers as *heterotopias* provides a novel lens for understanding how nurses assert professional identity and contribute to collective clinical decision‐making. The findings underscore the importance of preserving handovers as protected, *face‐to‐face* spaces that support clinical reasoning, mentorship and continuity of care. As digital and ‘silent’ reporting expand, safeguarding these relational and pedagogical functions remains essential to nursing practice in contemporary ICUs.

## Author Contributions

Conceptualization, methodology and data analysis: S.P. and E.P.; supervision: E.P.; validation: E.P. and F.T.; writing – original draft preparation: S.P. and M.M.; writing – review and editing: S.P., F.T., M.M. and T.A. All authors have read and agreed to the published version of the manuscript.

## Funding

The authors have nothing to report.

## Ethics Statement

Although the study involved healthcare professionals and did not involve patients or vulnerable populations, its design adhered to the ethical principles outlined in the Declaration of Helsinki, including voluntary participation, informed consent and protection of participant confidentiality. The study was approved (a) by the PhD programme at the Department of Social Anthropology, PANTEION University of Social and Political Studies in Athens, and (b) by the Institutional Research and Ethics Committee of the ‘ATTIKON’ hospital in Athens where the study was conducted (protocol number 7‐11/7/12, date 11/7/12).

## Consent

Informed consent was obtained from all participant healthcare professionals involved in the study.

## Conflicts of Interest

The authors declare no conflicts of interest.

## Data Availability

The data that support the findings of this study are available on request from the corresponding author. The data are not publicly available due to privacy or ethical restrictions.
